# A coarse-grained model for synergistic action of multiple enzymes on cellulose

**DOI:** 10.1186/1754-6834-5-55

**Published:** 2012-08-01

**Authors:** Andrea Asztalos, Marcus Daniels, Anurag Sethi, Tongye Shen, Paul Langan, Antonio Redondo, Sandrasegaram Gnanakaran

**Affiliations:** 1 Center for Nonlinear Studies, Los Alamos National Laboratory, Los Alamos, NM, 87545, USA; 2 Department of Physics, University of Notre Dame, Notre Dame, IN, 46556, USA; 3 Present Address: Department of Computer Science, Rensselaer Polytechnic Institute, Troy, NY, 12180, USA; 4 Computer, Computational, and Statistical Sciences Division, Los Alamos National Laboratory, Los Alamos, NM, 87545, USA; 5 Theoretical Division, Los Alamos National Laboratory, Los Alamos, NM, 87545, USA; 6 Present Address: UT-ORNL, Center for Molecular Biophysics and Department of Biochemistry, Cellular & Molecular Biology, University of Tennessee, Knoxville, TN, 37996, USA; 7 Bioscience Division, Los Alamos National Laboratory, Los Alamos, NM, 87545, USA; 8 Present Address: Biology and Soft Matter Division, Oak Ridge National Laboratory, Oak Ridge, TN, 37831, USA

**Keywords:** Cellulose degradation, Synergy, Exo-cellulase, Endo-cellulase, Agent-based model, Spatial heterogeneity

## Abstract

**Background:**

Degradation of cellulose to glucose requires the cooperative action of three classes of enzymes, collectively known as cellulases. Endoglucanases randomly bind to cellulose surfaces and generate new chain ends by hydrolyzing β-1,4-D-glycosidic bonds. Exoglucanases bind to free chain ends and hydrolyze glycosidic bonds in a processive manner releasing cellobiose units. Then, β-glucosidases hydrolyze soluble cellobiose to glucose. Optimal synergistic action of these enzymes is essential for efficient digestion of cellulose. Experiments show that as hydrolysis proceeds and the cellulose substrate becomes more heterogeneous, the overall degradation slows down. As catalysis occurs on the surface of crystalline cellulose, several factors affect the overall hydrolysis. Therefore, spatial models of cellulose degradation must capture effects such as enzyme crowding and surface heterogeneity, which have been shown to lead to a reduction in hydrolysis rates.

**Results:**

We present a coarse-grained stochastic model for capturing the key events associated with the enzymatic degradation of cellulose at the mesoscopic level. This functional model accounts for the mobility and action of a single cellulase enzyme as well as the synergy of multiple endo- and exo-cellulases on a cellulose surface. The quantitative description of cellulose degradation is calculated on a spatial model by including free and bound states of both endo- and exo-cellulases with explicit reactive surface terms (e.g., hydrogen bond breaking, covalent bond cleavages) and corresponding reaction rates. The dynamical evolution of the system is simulated by including physical interactions between cellulases and cellulose.

**Conclusions:**

Our coarse-grained model reproduces the qualitative behavior of endoglucanases and exoglucanases by accounting for the spatial heterogeneity of the cellulose surface as well as other spatial factors such as enzyme crowding. Importantly, it captures the endo-exo synergism of cellulase enzyme cocktails. This model constitutes a critical step towards testing hypotheses and understanding approaches for maximizing synergy and substrate properties with a goal of cost effective enzymatic hydrolysis**.**

## Background

Biofuel production from lignocellulosic materials is considered to be a promising option to substantially reduce the dependence on petroleum [1-3]. The conversion of lignocellulosic biomass (agronomic residues, paper wastes, energy crops) into ethanol consists of the extraction and pretreatment of cellulose from the biomass, hydrolysis (the enzymatic breakdown of crystalline cellulose fibers into monomer glucose) and finally the fermentation of glucose to ethanol. Current approaches mainly differ from one another in the method of pretreatment. Cost-competitive production of ethanol is currently prevented by the low efficiency of converting cellulose into glucose [4]. Greater efficiency may be achievable through improvements in hydrolysis.

Enzymatic hydrolysis of cellulose is a complex reaction. In the classical model, the heterogeneous catalytic cleavage of the glycosidic bond takes place on a crystalline cellulose surface and requires the cooperative action of three classes of aqueous enzymes, collectively known as cellulases. These are (i) endoglucanases, (ii) exoglucanases or cellobiohydrolases and (iii) β-glucosidases. Recently, it has been proposed that oxidative enzymes (monoxygenases) may also play a role in cleaving glycosidic bonds, although this new mechanism may be restricted to certain types of microbes [5]. It is widely accepted that endoglucanases cleave β-1,4-D-glycosidic bonds at random sites within both amorphous and crystalline polysaccharide chains, creating new chain ends on the cellulose surface [6-11]. Exoglucanases prefer to hydrolyze crystalline cellulose chains by acting on the free chain ends and releasing cellobiose units in a processive manner. Soluble cellobiose units are then converted into glucose by β-glucosidases. Consequently, these enzymes display strong synergy [12-18].

The classical chemical kinetics assumption of uniformly mixed systems does not hold in the case of enzymatic hydrolysis of cellulose fiber, as it is heterogeneous in nature. Such reactions are rather characterized by time-dependent rate constants and non-uniform concentration variation of reacting species. Although kinetic models [11,19-22] have been used to explain various features of the enzymatic hydrolysis of cellulose, they fail to account for spatial details of the cellulose substrate as well as the specificity of binding sites. Recently, Zhou and colleagues [23-25] proposed a “morphology-plus-kinetics rate equation approach” that explicitly captures the hydrolytic evolution of cellulose substrate. In addition, a kinetic model was developed based on population-balance equations in which a distribution of chains with different chain-lengths was explored [26,27]. Yet, these models give little insight regarding the action of cellulases at the molecular level.

It is imperative to develop spatial models of cellulose degradation because spatial effects such as enzyme crowding on the cellulose surface have been shown to lead to a reduction in hydrolysis rates. In order to account for the spatial heterogeneity of the system during cellulose hydrolysis, a cellular automata model [28] was developed to study the effect of different parameters such as enzyme binding and hydrolysis on the overall kinetics of cellulose by the cellulases. Alternatively, all-atom molecular dynamics (MD) simulations can provide details of molecular level events at high precision. Recent MD simulation studies [29-32] have proven effective for understanding enzyme-substrate binding, processivity and activity. However, because of length and time scale limitations, it is not currently possible to simulate the entire crystalline cellulose degradation process using all-atom MD simulations.

We have developed a coarse-grained stochastic model that captures the interaction of endo- and exo-cellulases with crystalline cellulose at a mesoscopic level. This model was specifically designed to improve our understanding of the molecular-level details of the enzymatic hydrolysis of crystalline cellulose. This paper introduces the basic framework and demonstrates how this model can be an effective and easily modifiable testing platform for new hypotheses based on experimental data on various cellulase components and substrate characteristics. By capturing the reactive nature of the cellulose substrate and the activities of non-complexed cellulases at the molecular level, this method forms a bridge between all-atom MD studies and deterministic reaction-rate approaches. To the best of our knowledge, it is the first model that is able to relate the synergetic action of multiple enzymes to molecular level details such as the hydrogen bond network of a cellulose substrate.

## Results and discussion

### Model development

The overall efficiency of the heterogeneous catalysis that occurs in the enzymatic hydrolysis of crystalline cellulose depends on factors such as adsorption, desorption, diffusion rates on the insoluble cellulose substrate, and processivity. In our model, catalysis is broken down into distinct parts related to different kinetic events (chemical reactions) performed by individual particles (enzymes). Specifically, we include the following reactive events: adsorption of cellulases on the solid cellulose substrate, inter-chain hydrogen bond breaking, hydrolysis of glycosidic bonds, and desorption of cellulases from cellulose. These reactions constitute the main elements of this model, and their realization is achieved by following and updating the state (based on certain predefined rules discussed below) of each individual particle in the system as it evolves in time. The actions of cellulases are modeled based on the most abundant endoglucanase (EG I) and the two cellobiohydrolases (CBH I and CBH II) secreted by the filamentous fungus *Trichoderma reesei*[33], as these three enzymes have been widely studied [10,11,34-36] and have been the target for improvement/design for efficient biodegradation [37,38].

#### Model of cellulose substrate

**Figure 1:**
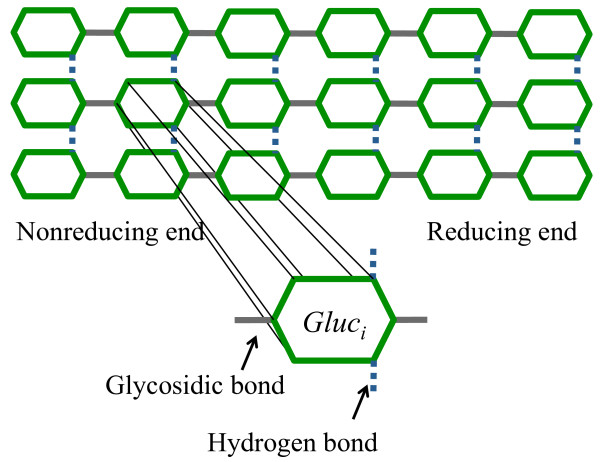
**Model for the cellulose surface composed of glucose.** At each time step, the stochastic simulation stores the intra- and inter-chain neighbors of each glucose unit. Additionally, each unit is characterized by a set of *P*_*m*_ parameters, *1 ≤ m ≤ 7*, listed in Table 1.

The cellulose surface layer (Figure 1) is modeled as a two-dimensional grid, consisting of multiple glucan chains, each of them having the same number of monomers, i.e., the degree of polymerization of glucan chains is the same. Glucose molecules are linked to each other through intra-chain covalent glycosidic bonds, while links between inter-chain glucose molecules correspond to multiple hydrogen bonds. In this representation, all chains are oriented such that the left side corresponds to the nonreducing end and the right side to the reducing end of the glucan chains. This chosen orientation represents the most commonly found form of crystalline cellulose in nature, cellulose Iβ [39]. The state of each glucose unit is defined by a set of seven binary parameters, *P*_*1*_*P*_*2*_, …,*P*_*7*_, listed in Table 1. Each row corresponds to one of the seven parameters characterizing one glucose unit. The values of the binary parameters (0 and 1) represent various conditions of one glucose unit.

**Table 1:** **State variables of a glucose unit**^**a**^

**P/Value**	**0**	**1**
*P*_*1*_	nonsoluble	soluble
*P*_*2*_	N.E.	R.E.
*P*_*3*_	uncovered	covered by endo
*P*_*4*_	uncovered	covered by exo-R
*P*_*5*_	uncovered	covered by exo-N
*P*_*6*_	not locked	locked by exo-R
*P*_*7*_	not locked	locked by exo-N

The first parameter, *P*_*1*_ informs whether the glucose unit belongs to the cellulose substrate (‘nonsoluble’) or is in the aqueous phase (‘soluble’). Initially, all glucose molecules are part of the cellulose surface (*P*_*1*_ = 0), and as the simulation progresses, they become soluble (*P*_*1*_ = 1) in the form of simple sugars: glucose, cellobiose or cellotriose. The length of the soluble oligomers can be easily modified.

The second parameter, *P*_*2*_ specifies whether the glucose unit constitutes the nonreducing end (NE) or the reducing end (RE) of a glucan chain. Also, when a glycosidic bond in the middle of a chain is hydrolyzed, two new ends are created, one new NE and one new RE.

Parameter *P*_*3*_ informs whether an endo-cellulase covers the glucose unit. Similarly, the values of parameters *P*_*4*_ and *P*_*5*_ indicate whether an exo-cellulase hydrolyzing from the reducing end (exo-R) or an exo-cellulase hydrolyzing from the nonreducing end (exo-N) covers the glucose unit. At any time, only one cellulase is allowed to cover a specific glucose, during which glycosidic and hydrogen bonds may be cleaved or broken. A glucose unit, which is not covered by any cellulases, may become *locked* by a processive cellulase, exo-R or exo-N. This is specified by parameters *P*_*6*_ and *P*_*7*_, respectively. A locked glucose unit only facilitates the binding of the locking exo-R (exo-N); it does not constitute an available binding site for any other cellulases, nor can its glycosidic or hydrogen bonds be cleaved or broken, until the locking cellulase binds directly to it.

#### Model of cellulase with endo-activity

**Figure 2:**
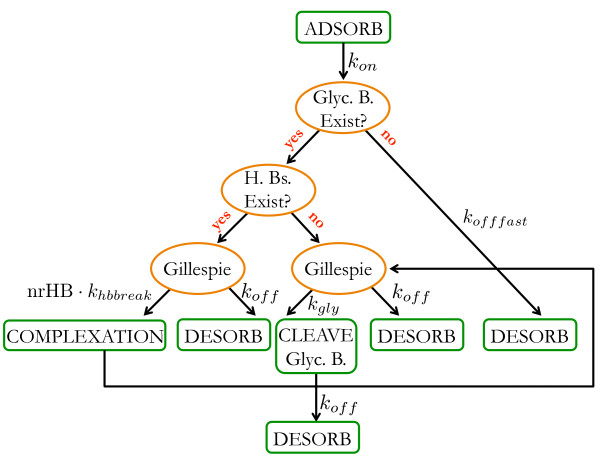
**Flowchart presenting the actions of an endo-cellulase. The actions are: **adsorption onto the cellulose surface (ADSORB), breaking hydrogen bonds (BREAK H. Bs.), hydrolyzing the glycosidic bond (CLEAVE Glyc. B.), and desorption from the cellulose (DESORB). Green rectangles denote chemical reactions (events) and orange ellipses denote branching points. Abbreviations: ‘Glyc. B.’ covalent glycosidic bond; ‘H. Bs.’ hydrogen bonds; ‘nrHB’ number of hydrogen bonds present between the monomers covered by the cellulase.

Cellulases with endo-activity (referred to as endo-cellulases or simply endo) are modeled through a set of interactions between the cellulose surface and among themselves. A detailed description of the actions of adsorbed endo-cellulases is presented in Figure 2, while Table 2 lists the parameters that determine their overall activity. Additionally, a state parameter is used to specify whether the cellulase is adsorbed to the substrate or is in solution. In the future, we plan to incorporate another state parameter to describe a decrystallization step that “prepares” the substrate for productive binding.

**Table 2:** Rate constants characterizing endo-cellulases

**Nomenclature**	
*k*_*on*_	adsorption rate constant
*k*_*off*_	desorption rate constant
*k*_*offfast*_	a higher desorption rate constant than *k*_*off*_
*k*_*hbbreak*_	rate constant for breaking a single hydrogen bond
*k*_*gly*_	rate constant for hydrolyzing a glycosidic bond

Endo-cellulases, once adsorbed to the cellulose surface, can break inter-chain hydrogen bonds, hydrolyze glycosidic bonds and desorb from the substrate into solution. Each of these chemical reactions is essentially a Poisson process that takes place at a specific, constant rate defined by the propensity function of that reaction [40].

**Figure 3:**
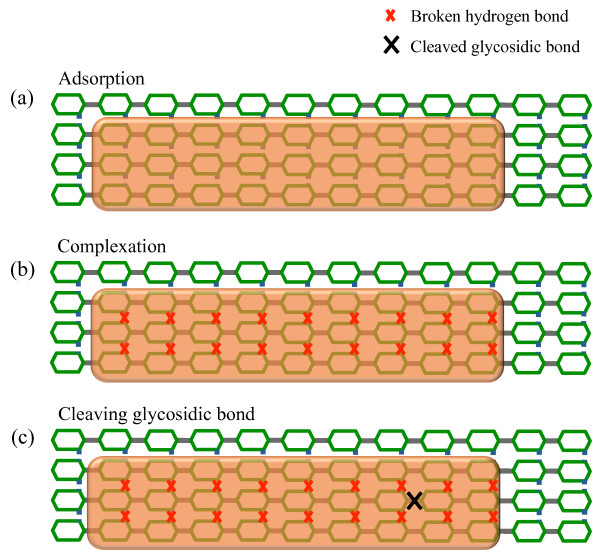
**Interactions between endo-cellulase and cellulose crystals.** Schematic representation of (a) an endo-cellulase adsorbed onto the cellulose surface, (b) hydrogen bonds breaking between the monomers covered by the enzyme and (c) hydrolysis of the glycosidic bond.

Each endo-cellulase adsorbs to a randomly chosen *available site*. A *site* is a set of glucose units, as presented in Figure 3a, consisting of nine consecutive glucose units in three neighboring chains, for a total of 27 adjacent glucose units [35]. However, we assume that just a length of 4 glucose units in the middle chain is enough for it to form a productive complex. This choice was motivated by the empirical studies of Claeyssens et al. [41] and Biely et al. [42] who argued that the substrate binding site of EG I is an extended one, consisting of four sugar binding subsites with a catalytic group located in the middle. A site is *available* if all monomers in the middle glucan chain belong to the cellulose substrate and none of the twelve monomers is covered by another enzyme nor are they locked. Desorption of the cellulase might take place at any time. If the glycosidic bond in the *catalytic region* is already hydrolyzed, the cellulase desorbs into solution within an exponentially distributed time interval with rate parameter *k*_*offfast*_ (see Figure 2). The catalytic region of an endo-cellulase is considered to be the glycosidic bond between the second and third glucose units in the middle chain covered by the enzyme, shown in Figure 3c. The hydrolysis of the glycosidic bond can only take place after all inter-chain hydrogen bonds between the covered glucose units are broken (Figure 3b). The time it takes to break the hydrogen bonds is proportional to the number of bonds that need to be broken, denoted by ‘nrHB’ in Figure 2. If there is at least one hydrogen bond that needs to be broken, the cellulase breaks it or desorbs into solution. Similarly, if all hydrogen bonds have already been broken, as shown in Figure 3b, the cellulase either hydrolyzes the glycosidic bond at the catalytic region or desorbs into solution. These decisions are implemented using Gillespie’s algorithm [40]. Hydrolysis of a glycosidic bond is always followed by desorption of the cellulase within an exponentially distributed time with rate parameter *k*_*off*_.

#### Models for cellulases with exo-activity

**Figure 4:**
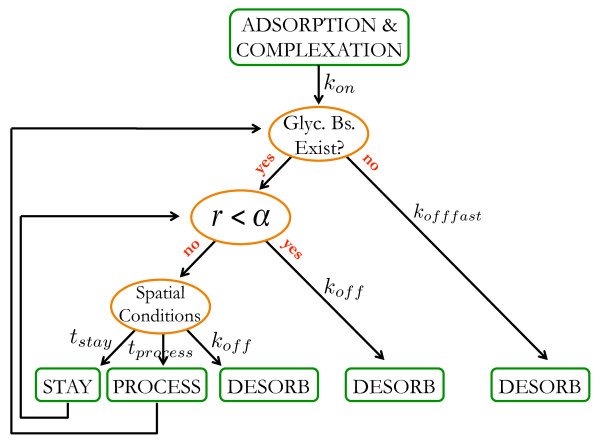
**Flowchart presenting the actions of an exo-cellulase after adsorption to the cellulose surface.** Chemical reactions are denoted by green rectangles; orange ellipses denote branching points. Abbreviations are the same as used in Figure 2. Additionally, *r* is a uniformly, distributed random variable between *0* and *1*.

Cellulases with exo-activity can be of two types: cellulases hydrolyzing the glucan chain from its reducing end (referred to as exo-R cellulase or simply exo-R), and cellulases hydrolyzing the glucan chain from its nonreducing end (referred to as exo-N cellulase or simply exo-N). The interactions between adsorbed exo-cellulases and the cellulose surface are shown in Figure 4, while Table 3 lists the relevant parameters that determine the overall activity of exo-cellulases. Additionally, a state parameter is used to specify whether the cellulase is adsorbed to the substrate or is in solution. Again, we intend to consider an additional state parameter to describe the decrystallization step in future models.

**Table 3:** Rate constants and relevant parameters for characterizing and exo-cellulase

**Nomenclature**	
*k*_*on*_	adsorption rate constant
*k*_*off*_	desorption rate constant
*k*_*offfast*_	a higher desorption rate constant than *k*_*off*_
*α*	probability for the exo-cellulase to desorb from cellulose
*t*_*hbbreak*_	time for an exo-cellulase to break a single inter-chain hydrogen bond
*t*_*move*_	time for an exo-cellulase to hydrolyze a glycosidic bond and slide one cellobiose unit along the glucan chain
*t*_*stay*_	time for an exo-cellulase to remain at a certain location

Exo-cellulases, once adsorbed to the cellulose surface, can break hydrogen bonds, hydrolyze glycosidic bonds, slide along a chain, and desorb from the cellulose into solution. For simplicity, in the following description we restrict ourselves to actions carried out by an exo-R cellulase. The ones carried out by an exo-N cellulase are essentially the same.

**Figure 5:**
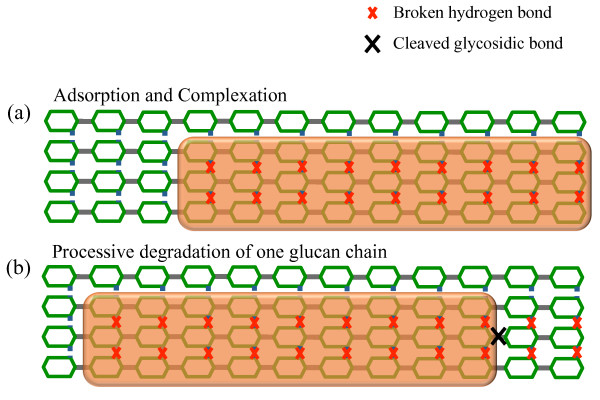
**Interactions between exo-cellulase and cellulose crystal.** Schematic representation of (a) an exo-R cellulase adsorbed to the cellulose surface followed immediately by the breaking of hydrogen bonds between the monomers covered by the cellulase and (b) the processivity of the glucan chain by an exo-R cellulase; it comprises the hydrolysis of the glycosidic bond and cellulase directed movement along the chain.

The adsorption site of an exo-R cellulase consists of nine consecutive glucose units in three neighboring chains, for a total of 27 adjacent glucose units (shown in Figure 5a), subject to the condition that the middle glucan chain has a reducing end. This choice has been motivated by the three-dimensional structure of the catalytic domain of CBH I from *T. reesei*[34,43]. This catalytic site resides within a relatively long (~50 Å) cellulose binding tunnel holding ten glucose molecules, out of which three—near the outlet—form the product binding sites. As cellobiose is the main product released by CBH I [16,44], the exo-cellulase in our model has only two product binding sites, which, for the substrate, translates into a total of nine sugar binding sites. The adsorption site for an exo-cellulase is considered to be *available* if all monomers in the middle glucan chain belong to the cellulose substrate and none of the 27 monomers is covered by another enzyme nor are they locked.

During adsorption, the inter-chain hydrogen bonds of the glucose units beneath the cellulase are instantly broken (Figure 5a). Similar to an endo-cellulase, desorption of an exo-cellulase from the cellulose sheet can take place at any time. If any of the glycosidic bonds between the glucose units covered by the cellulase in the middle glucan chain is already hydrolyzed, the cellulase desorbs from the surface within an exponentially distributed time interval with rate parameter *k*_*offfast*_*.* This assumption was built into the model in order to account for the continuity of the chain entering the tunnel of CBH I. The frequency of cellulase dissociations from the substrate is set by a probability α. Results of high speed AFM measurements [45] indicate that once the cellulase is adsorbed to a free end, it continues to process the chain until it reaches the end of the chain. In this light, α is usually set to a relatively low value. The ‘Spatial Conditions’ branching point in Figure 4 checks whether the cellulase can further hydrolyze the glucan chain. When another cellulase obstructs its diffusion, it may stay in place for a time of length *t*_*stay*_. It may also desorb within an exponentially distributed time with parameter *k*_*off*_, if glucose units are missing from the middle chain or if it has reached the nonreducing end of the chain (and similarly for the exo-N).

The processive movement of exo-cellulases is modeled as a two-step process: *(i)* the cellulase hydrolyzes the glycosidic bond positioned at the *active site*, followed by *(ii)* its sliding along the processed chain by one cellobiose unit, instantly breaking inter-chain hydrogen bonds [34] (Figure 5b). The active site of an exo-R is considered to be the glycosidic bond between the second and third glucose unit from the reducing end of the middle, processed glucan chain. If the two glucose units in front of the cellulase belong to the substrate, and none of the six glucose units in front of the cellulase (two consecutive ones in three chains) are occupied by any other cellulases, these six monomers become locked, implying that they could be covered by the exo-cellulase. Locked glucose units are not considered available binding sites for endo– or exo–cellulases. The processing time of exo–cellulases is calculated as *t*_*process*_ *= t*_*move*_ *+ t*_hbbreak_*nrHb, where *t*_*move*_ specifies the time during which the cellulase hydrolyzes the glycosidic bond at the active site *and* slides along the middle glucan chain by one cellobiose unit. Here *nrHb* is the number of present hydrogen bonds between the six locked monomers. The same strategy is employed for exo-N except that the processivity is along the opposite direction towards the left-hand side. Furthermore, an exo-cellulase is never allowed to productively bind to chains in the middle of the cellulose surface. The exo-R cellulases are only allowed to productively bind to a free reducing end, while the exo-N cellulases are only allowed to productively bind to a free non-reducing end.

#### Algorithm for time evolution

**Figure 6:**
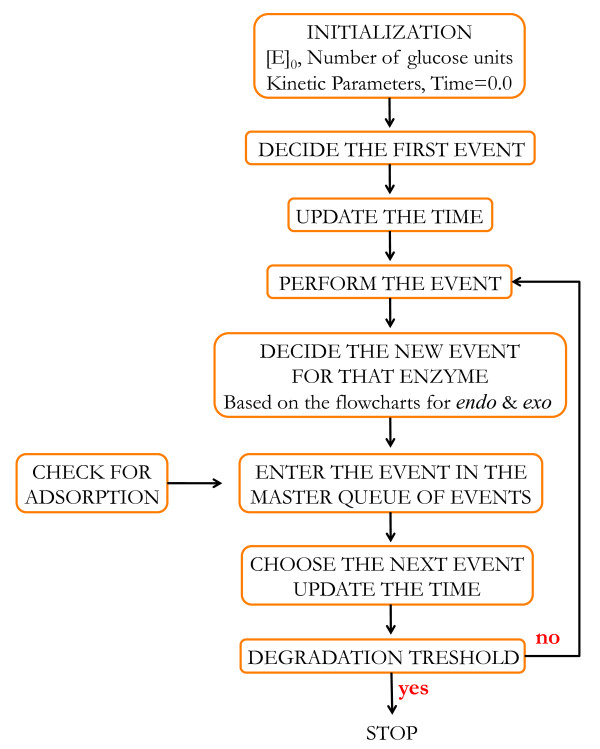
**Simulation timeline.** Here an ‘EVENT’ may refer to any chemical reaction that involves a cellulase (adsorption, desorption) or is catalyzed by a cellulase (e.g., inter-chain hydrogen bond breaking, hydrolysis of glycosidic bond).

The outline of the overall simulation is sketched in Figure 6. Time is measured in seconds and is advanced in a continuous and asynchronous manner. The simulation starts with the adsorption of an individual cellulase (endo or exo) and it proceeds following the flowcharts presented in Figure 2 (for endo-cellulase) or Figure 4 (for exo-cellulase). Each individually adsorbed cellulase is followed separately over the course of the simulation. While in solution, they all compete with each other for adsorption, and once adsorbed, the selection of the chemical reactions they are involved in follows a well-defined, rule-based schematic (see below) that finally leads to the hydrolysis of the entire cellulose substrate. However, Gillespie’s algorithm [40,46,47] plays a crucial part in the simulation, as many chemical reactions, once selected by the rule-based scheme, are modeled as Poisson processes and therefore are implemented using this algorithm. Gillespie’s algorithm is a Monte Carlo technique that allows one to sample the ensemble of trajectories for a set of biochemical reactions. It models chemical reactions as a stochastic process and remains valid for low copy numbers of reactants. Here we implement the direct version of this method [46].

Chemical reactions that involve endo-cellulases (adsorption, desorption) or are catalyzed by endo-cellulases (inter-chain hydrogen bond breaking, hydrolysis of glycosidic bond) are modeled as Poisson processes. This choice has been motivated by the knowledge of the specific reaction rate constants.

A different modeling approach is taken in the case of exo-cellulases. While adsorption of exo-cellulases to the cellulose substrate is modeled as a Poisson process, each adsorbing event is combined with an instantaneous structural transformation of the surface as hydrogen bonds below the cellulase are assumed to break at the very moment of adsorption. The processivity of exo-cellulases is defined through specific rules: when certain spatial conditions are met, the cellulase slides along the glucan chain by one cellobiose unit leaving behind a soluble cellobiose and instantly changing the cellulose surface (breaking intact hydrogen bonds) beneath itself. We assume that each exo-cellulase has the same, constant processing velocity so the only difference in processing times of one exo-cellulase from another originates in the number of intact hydrogen bonds covered by respective cellulases. Finally, desorption of exo-cellulases is also modeled as a Poisson process with a constant rate parameter.

Each chemical reaction is modeled as a discrete event occurring instantaneously while the state of the system remains unchanged between two consecutive events. The events associated with each of the adsorbed cellulases are stored in a priority queue, here referred to as the *master queue of events* (Figure 6)*.* They are sorted by the simulated time at which they should occur. The simulation runs until the surface degrades to a specific *degradation threshold*, which is usually set to 100%, or the point at which the substrate is not able to adsorb more enzyme particles.

#### Simulation parameters

The values of the kinetic parameters we employed are listed in Table 4. The values of *k*_*on*_ and *k*_*off*_ were determined based on the adsorption equilibrium constants, which for both types of cellulases were equal to *10*^*3*^ *M*^*-1*^. The fast desorption rate constant (*k*_*offfast*_) was always one order of magnitude larger than *k*_*off*_. The values of the hydrolysis rate constants for both endo- and exo-cellulases (*k*_*gly*_) were the ones estimated by Zhang and Lynd [22] and accordingly, the rate constant for endo-cellulases was set at fivefold that used for exo-cellulases [11,48]. From there we obtain *t*_*move*_ *= 12 s*; *t*_*stay*_ was set to the same value. The probability *α* was set to *0.1* and the time to break a hydrogen bond to *10*^*-12*^ s [49]*.* In the results presented in the next section, we did not include hydrogen bond reformation, but this is a reaction that can easily be included in this model.

**Table 4:** Input parameters used in simulations unless otherwise stated

**Name**	**Notation**	**Endo**	**Exo-R/Exo-N**
Molecular weight^b^	*μ*	52500 g/mol	63500 g/mol
Adsorption rate constant	*k*_*on*_	100 (sM)^-1^	10^4^ (sM)^-1^
Desorption rate constant	*k*_*off*_	0.1 s^-1^	10 s^-1^
Higher desorption rate constant	*k*_*offfast*_	1 s^-1^	100 s^-1^
Glycosidic bond hydrolysis^c^	*k*_*gly*_	0.35 s^-1^	0.0846 s^-1^
Hydrogen bond breaking^d^	*k*_*hbbreak*_	10^12^ s^-1^	10^12^ s^-1^

Table 4 also includes the molecular weights of EG I and CBH I used in the simulation. The molecular weights of glucose (*μ = 180.15588 g/mol*) and anhydroglucose (*μ = 162.1406 g/mol*) molecules are essential in the calculation of the soluble sugar concentration. The cellulose substrate is composed of five glucan chains, each of them having 4000–5000 glucose monomers. The area of a cellobiose unit [11] was set to *A*_*G2*_ *= 5.512 × 10*^*-19*^ *m*^*2*^. The initial (molar) enzyme concentration was calculated from the number of cellulases present in the system and the volume of the system, *V = Sd*, where *S* is the cellulose surface area in m^2^ and *d* is of the order of μm. In most of the runs the initial enzyme concentration was set to be *[E]*_*0*_ *= 2 μM*. In the following section, unless stated otherwise, we used the parameter values listed in Table 4 and the above enumerated initial conditions.

The output consists of the time evolution of the concentrations of glucose, cellobiose and cellotriose present in the aqueous phase, the adsorption density, and the number of available binding sites per gram of cellulose. Results were averaged over 10 replicas of the system. The conversion is quantified by counting the number of all the soluble glucose molecules including those in cellobiose and cellotriose and dividing that by the initial number of glucose molecules.

### Hydrolysis by endo-cellulases

**Figure 7:**
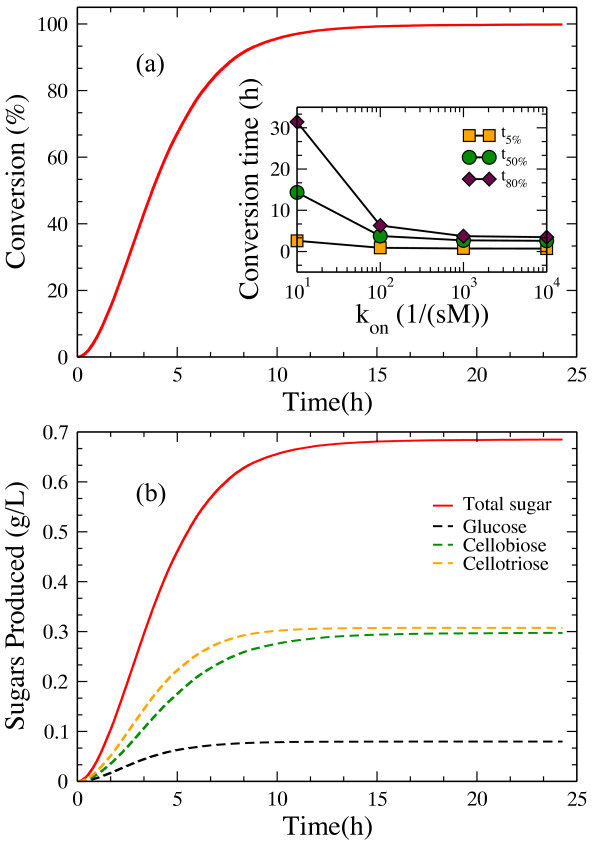
**(a) Hydrolysis by endo-cellulases.** Time course of hydrolysis by endo-cellulases.The inset shows the variation in the conversion time needed to degrade 5%, 50% and 80% of the substrate as a function of the adsorption rate constant *k*_*on*_. (**b**) Total sugar production and individual monomer and oligomer component distribution over time.

First we simulate and analyze the hydrolysis of a crystalline cellulose layer solely by endo-cellulases. Figure 7a shows the timeline of percent cellulose degradation. After an initial slow hydrolysis phase, the simulation results agree qualitatively with published experimental results [50]. Using parameter values listed in Table 4, our model reproduces well the observed experimental hydrolysis time scales. As expected, the time it takes to convert a given percent of the substrate decreases as *k*_*on*_ increases (Figure 7a inset). Similarly, the relative production of soluble oligosaccharides shown in Figure 7b agrees well with the experiments [11]. Cellobiose is the major type of soluble sugar, both in experiments and in our model. In our model, the final molar ratio between glucose and cellobiose is close to 1:10, while the final molar ratio between cellotriose and cellobiose is close to 7:10. In experiments, the glucose concentration is observed to be higher than the cellotriose concentration, while our model shows the opposite. Our model assumes that cellulose oligomers of length less than 4 enter solution and none of the modeled cellulases can digest them after they enter solution phase. This simplified assumption leads to disagreement with experiments.

**Figure 8:**
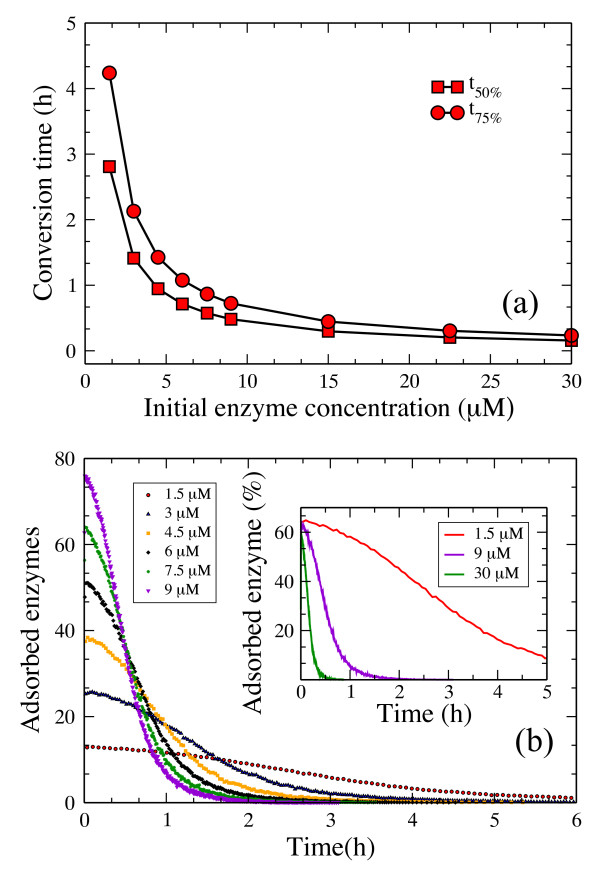
**(a) Effect of initial concentration of endo-cellulase on hydrolysis of cellulose.** Change in conversion time as the initial enzyme concentration is varied.(**b**) Time course of enzyme adsorption. The inset shows the percentage of adsorbed enzymes as a function of time. The cellulose layer is composed of five glucan chains, each of them composed of 5000 glucose units.

The effect of varying the initial *[E]*_*0*_ cellulase concentration upon conversion times is plotted in Figure 8a. As the enzyme concentration increases, the gap between the time to convert 50% and 75% of the cellulose substrate decreases. At high enzyme loading *(> 22 μM*), the conversion time reaches a constant value as the substrate is saturated by adsorbed enzymes: already bound enzymes mutually obstruct the adsorption of additional enzymes onto the surface. As the substrate is reduced over time, the number of available binding sites also decreases, thus fewer and fewer enzymes are able to bind to the surface. This trend is captured in Figure 8b. Cellulases quickly adsorb onto the surface at early times in the hydrolysis process (not shown because of the small time scale), after which their number follows a Poisson decay, and desorption is modeled as a Poisson process. The inset from Figure 8b shows the decrease in bound enzyme percentage as the initial enzyme concentration increases. Although this decrease is relatively small, it underlines the finite size effect of the substrate, which is consistent with Figure 8a.

**Figure 9:**
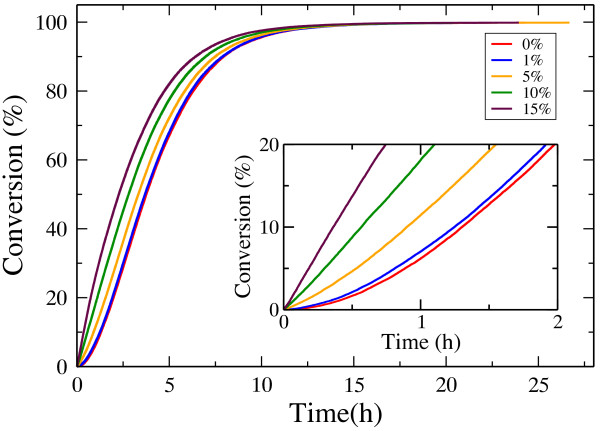
**Hydrolysis by endo-cellulases for imperfect crystals.** Time course of hydrolysis by endo-cellulases when various glycosidic bond percentages are initially cleaved in the cellulose layer. The inset shows the same curves for a short time scale.

Although the simulation results show good agreement with the experiments, the lag phase observed in the hydrolysis curve (Figure 7a) is unexpected, as it was not observed in any bulk measurements involving enzymatic hydrolysis of cellulose. It was, however, observed during acid hydrolysis of bacterial cellulose [51] and during anaerobic bacterial digestion of cellulose [52]. The possible reasons behind the occurrence of the lag phase are various. *(i)* At the beginning, the random glycosidic bond cleavages are too far from each other to release glucose, cellobiose or cellotriose, as this requires a finite amount of time for cellulases to revisit the neighborhood of a cleaved bond. *(ii)* The model used here does not take into account enzyme diffusion [53], which could accelerate the ability of the enzyme to locate the neighborhood of a cleaved glycosidic bond. *(iii)* In contrast to a realistic cellulose crystal surface with impurities and pre-existing broken inter- and intra-chain bonds, the simulated initial cellulose surface is perfectly regular and fully crystalline. Already, with only 5% of bonds hydrolyzed, the substrate becomes highly irregular (Shishir Chundawat, Personal Communication). In order to observe the effect of these irregularities, simulations were performed with an initial percentage of broken glycosidic bonds. Figure 9 shows how the initial slow hydrolysis phase diminishes as the initial cellulose substrate becomes more and more irregular.

Even though we obtain reasonable qualitative agreement, it cannot be simply improved by just using a few experimentally determined kinetic parameters. Since our model is a detailed one, there are several other parameters that need to be optimized as well to get quantitative correspondence. For example, when we use the experimentally determined rates for adsorption (k_on_ = 4.2 * 10^4^ s^-1^- based on association constant K_a_ = 1.4*10^6^ M^-1^ s^-1^[54] and k_off_[55]) and desorption (k_off_ = 0.03 M^-1^ s^-1^[55]) for EG-I from cellulose crystals, we observe that the enzyme hydrolyzes the cellulose crystals completely in under 15 hours. Other physical reasons may also contribute. One of the reasons for the fast processing rate observed in the model is because we only process two-dimensional crystals of cellulose, while cellulose crystals are three-dimensional in nature. In three-dimensional crystals, there are multiple layers of cellulose chains in the crystal, and not all of the glycosidic bonds are available as substrate for the enzyme to process from the beginning. Rather the inner layers of the crystal are available as substrate only after the layers above them are partially processed. Another reason could be that the rate constant for decrystallization (or chain separation) of cellulose crystals by the enzymes is based on the theoretical timescales for breaking of hydrogen bonds in an aqueous environment, which is quite rapid.

### Hydrolysis by exo-cellulases

**Figure 10:**
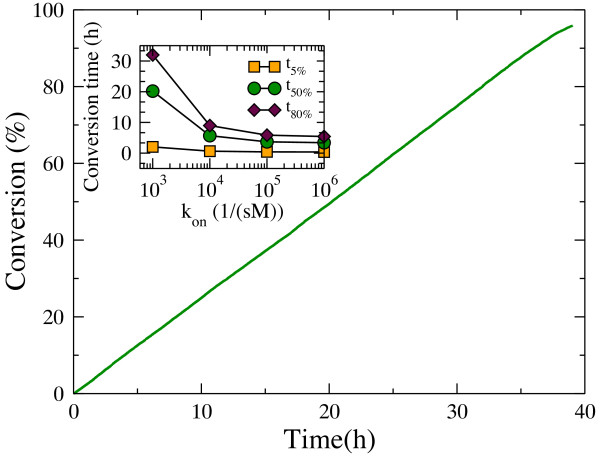
**Time course of hydrolysis by exo-R cellulases.** The adsorption rate constant was set to 10^3^ 1/(sM)). The inset shows the variation in the conversion time needed to degrade 5%, 50% and 80% of the substrate as a function of the adsorption rate constant *k*_*on*_.

The hydrolysis profile produced by exo-cellulases is shown in Figure 10. The cellulose conversion is constant over time, a feature that has not been observed in experiments [16,50]. As expected, the time it takes to convert a given percent of the substrate decreases as *k*_*on*_ increases (Figure 10, inset).

**Figure 11:**
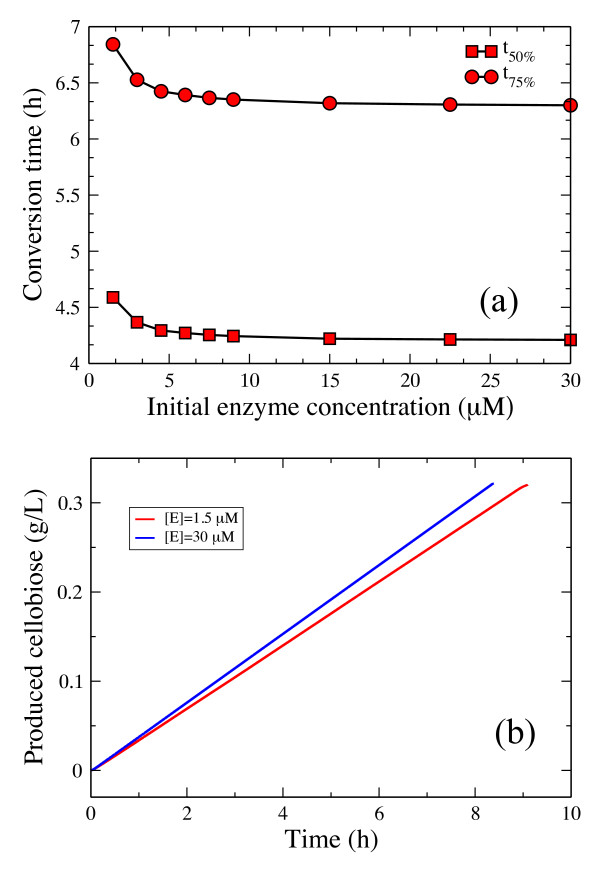
**(a) Effect of initial concentration of exo-cellulases.** Change in convencion time as initial enzyme concentration is varied(**b**) Sugar production over time. System parameters are: *N* = 25000 glucose units, *k*_*on*_ = 10^5^ 1/(sM), *k*_*off*_ = 100 1/s.

The effect of varying the initial *[E]*_*0*_ enzyme concentration upon conversion times is plotted in Figure 11a. In contrast to Figure 8a, the substrate becomes saturated by exo-cellulases at lower enzyme concentrations than we observed in the case of endo-cellulases. This is because the number of free chain ends always remains small compared to the number of enzyme particles in solution. Figure 11b shows that the only sugar produced by exo-cellulases is cellobiose. Experimental results, however, report the production of both glucose and cellotriose along with cellobiose, although cellobiose is the major product [16,50]. The relatively constant processive speed of exo-R cellulases along the glucan chain explains the constant hydrolysis rate observed in Figure 10 and the non-decreasing gap between the two curves in Figure 11a.

The relatively constant processing time of the exo-cellulases is the result of using a simple coarse-grained description of processing time for exo-cellaloses since no rates have been measured for specific events associated with processivity. Values in these calculations are set such that k_on_, k_off_ >> 1/t_move_ for exo-cellulases. Thus the binding of the enzyme to the substrate is at equilibrium. These parameters can be easily adjusted to match up with any forthcoming experimental observations. In addition, the total concentration of the substrate (reducing ends for exo-R or non-reducing ends for exo-N) does not change with time until the whole chain is processed. This ensures that the concentration of enzyme-bound substrate remains nearly constant with time for the exo-cellulases resulting in a nearly constant rate of processing until the end. We expect these effects to be reduced in three-dimensional crystals of cellulose in which multiple layers of cellulose chains have to be processed by the exo–cellulases as the chains get hydrolysed in a staggered fashion in these crystals. It has also been reported [56] that cellulose-binding modules bind to insoluble non-crystalline cellulose with a 10-20-fold greater affinity than to cello-oligosaccharides and/or soluble polysaccharides. Future expansion of this model will incorporate a non-constant adsorption rate of enzymes that would depend on the length of the cello-oligosaccharides; this will bring further complexity to the model. In addition, incorporation of stochasticity in the processing of the cellulose chain by the exo-cellulases and better estimates for rates of decrystallization of the cellulose crystal could lead to better agreement with experimental hydrolysis rates.

### Hydrolysis by endo- and exo-cellulases

**Figure 12:**
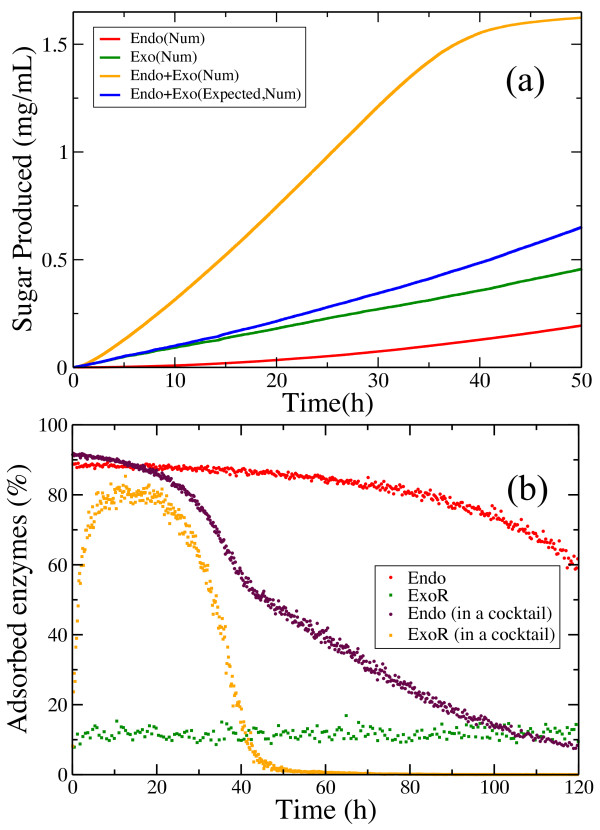
**Endo-exo synergy.** (**a**) Comparison of the time course of sugar production when cellulose is degraded only by endo-cellulases (red curve), only by exo-R cellulases (green curve), or when the two types of enzymes were acting together (orange curve). The sum of the red and green curves (blue curve) yields lower sugar production than in the case when the substrate is converted simultaneously by endo- and exo-cellulases. (**b**) Adsorbed enzyme percentages during hydrolysis when the cellulases are acting alone (red and green dots) or in a mixture (maroon and orange dots).

In order to test whether our model reproduces the experimentally observed endo-exo synergy, we used experimental data reported by Eriksson and colleagues (see Figure 1A[50]) and modified the kinetic rate constants for both types of cellulases by fitting the model single enzyme hydrolysis curves to the experimental data. The initial enzyme concentration was set to *[E]*_*0*_ *= 1.5 μM* and the cellulose concentration to *10 g/L*[50]. Numerical results show the endo-exo synergy (Figure 12a) and the time scale is the same as observed experimentally [50]. It should be kept in mind that the substrate in that experimental work is steam-pretreated spruce (lignocellulose), not pure cellulose. However, it is encouraging that we do observe similar behavior. Sugar production is higher when exo-R cellulases hydrolyze the cellulose surface in the presence of endo-cellulases when compared to the sum of their conversions achieved alone.

The increase of free chain ends produced by endo-cellulases is the primary source of the endo-exo synergy observed in the model. Figure 12b illustrates how this effect contributes to a large increase in the percentage of adsorbed exo-cellulases. This percentage is constant when exo-cellulases degrade the substrate alone, while in the presence of endo-cellulases it grows to higher values, contributing to a fast and efficient degradation of the substrate.

**Figure 13:**
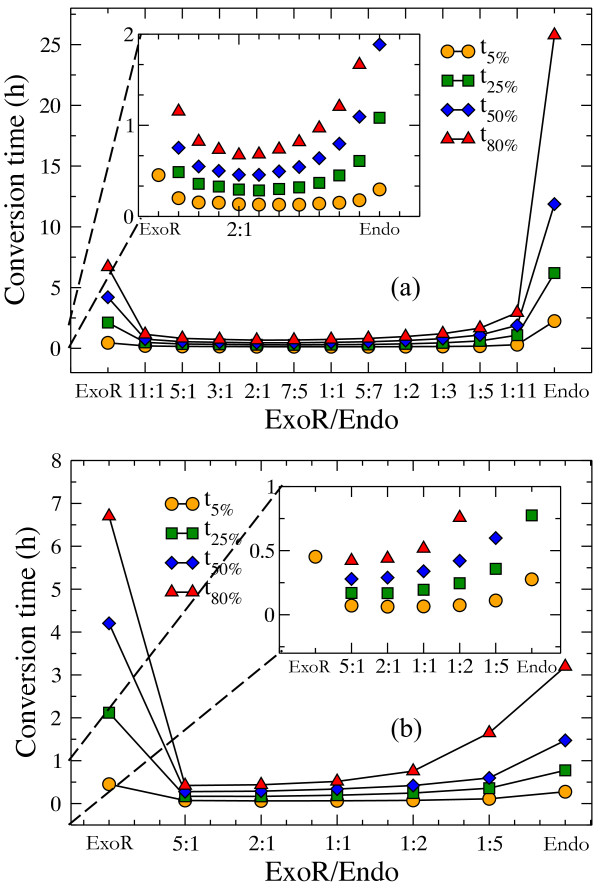
**Effect of composition of endo-exo mixture.***(a) k*_*on*_*(endo) = 10(sM)*^*-1*^*, k*_*off*_*(endo) = 0.01 s*^*-1*^ and (b) *k*_*on*_*(endo) = 100(sM)*^*-1*^*, k*_*off*_*(endo) = 0.1 s*^*-1*^*.* In both cases *k*_*on*_*(exo-R) = 10*^*4*^*(sM)*^*1*^, *k*_*off*_*(exo-R) = 10s*^*-1*^. (*N = 25000* glucose units, total cellulase concentration is *2 μM*).

Results regarding synergism between pure *Trichoderma* cellulases [14,57] showed that the endo-exo synergy depends on the ratio of the concentrations of the individual enzymes. Here, we tested whether our model qualitatively reproduces this observation by comparing conversion times—the time to degrade 5%, 25%, 50% or 80% of the substrate—for various exo-R/endo ratios. Using the hydrolysis rates listed in Table 4, we consider two cases: *i)* the overall hydrolysis of cellulose by endo-cellulases takes place at a slower rate than the overall hydrolysis of cellulose by exo-cellulases (Figure 13a); *ii)* the overall hydrolysis by exo-cellulases is set to be slower than that by endo-cellulases (Figure 13b). As the rate-limiting step in the model is the adsorption of cellulases onto the substrate, we attain this by varying the *k*_*on*_ adsorption rate constant while fixing the equilibrium constant of each of the cellulases. In both cases the substrate conversion time has a minimum at a specific ratio of the concentrations of the individual enzymes. For the first case (see Figure 13a) the optimal exo-R/endo ratio is 2:1, while for the second case (see Figure 13b) this ratio is 5:1. These minimum conversion times in both cases are much smaller than the conversion times obtained in single cellulase runs. These optimal ratios were obtained for a perfectly regular cellulose substrate; however, as pointed out earlier [14,57], the optimal experimental ratio is strongly dependent on the characteristics of the substrate. For example, on filter paper [57] the optimal exo-R/endo ratio was found to be 74:26 when the total enzyme concentration was *1 μM* and 90:10 when the total enzyme concentration was *10 μM.*

### Sensitivity analysis

**Figure 14:**
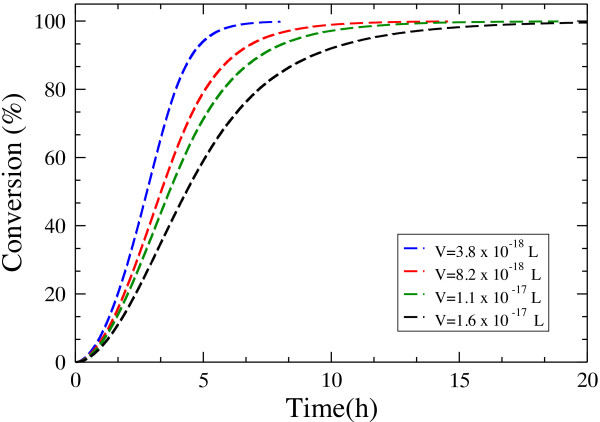
**Volume dependence of the time course of hydrolysis by endo-cellulases (*N***_***E***_ **= 10)**.

We have also carried out a sensitivity analysis to evaluate the relative importance of some of the physical quantities involved in the simulations. First, we studied the *volume dependence* of the overall hydrolysis. The reaction volume, *V*, determines the adsorption rate and therefore it determines the amount of adsorbed cellulases, affecting the rate of the overall hydrolysis. The simulation behaves as expected: a smaller volume results in higher adsorption rates, leading to faster hydrolysis (Figure 14).

**Figure 15:**
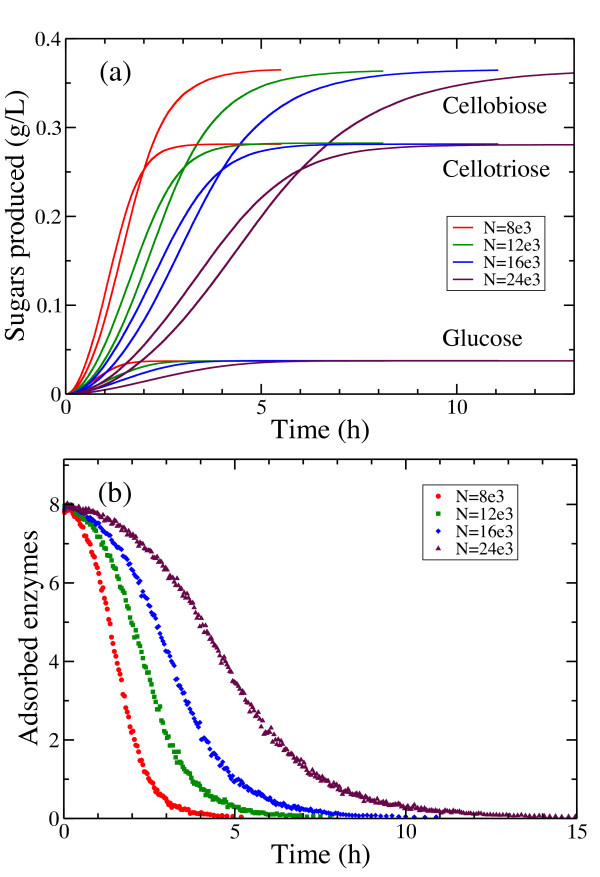
**The effect of varying the substrate size.** (**a**) Time course of sugar production and (**b**) of adsorbed cellulases when cellulose is hydrolyzed by endo-cellulases (*N*_*E*_ = 10).

Next, the absolute *size of cellulose surface* was varied to determine the effect of the size of the simulation cell (Figure 15a). It is reassuring to see that the size of the substrate does not have any effect on the oligomer distribution. The amount of glucose, cellobiose and cellotriose increases as the substrate becomes larger, but their relative concentrations are not affected by the substrate. A larger substrate needs more time to be degraded by the same amount of cellulases. The amount of adsorbed endo-cellulases is plotted as function of time in Figure 15b. The system size does not qualitatively change the number of adsorbed cellulases, it only affects the overall hydrolysis time, and as such the curves get shifted towards larger time scales.

## Conclusions

In an effort to complement both all-atom molecular dynamics and coarse-grained simulation tools, we have developed a an agent-based the dynamical, functional model capturing the surface chemical reaction of cellulose hydrolysis by enzymes at the molecular level. This model accounts for heterogeneous enzymatic hydrolysis reactions occurring on the substrate surface (a reaction taking place in dimensions less than three), and incorporates key factors controlling it that are different from those in an aqueous environment. The catalysis process is broken down into distinct parts related to different kinetic events carried out by individual particles. These events are essentially chemical reactions taking place on the surface of cellulose (adsorption, breaking inter-chain hydrogen bonds, cleaving glycosidic bonds, desorption) and constitute the main elements of this model. Reactions are monitored by following and updating the state (based on a set of predefined rules) of each individual particle in the system. Simulation results showed good qualitative agreement with experimental data.

The agreement with experimental data can be improved by obtaining better experimental estimates of the parameters in Table 4 and by extending the current model to three dimensions. Initial experiments that can greatly benefit the model are those that probe the kinetics of different steps for the individual domains of the cellulases (Carbohydrate Binding Domain and Catalytic Domain) separately. These experiments need to quantify the binding affinities, *k*_*on*_ and *k*_*off*_. Then similar measurements on the entire cellulases for binding of the same substrate will help to verify the role of individual domains and provide a measure of productive and non-productive binding. These measurements need to be carried out for pure cellulose substrates of different shapes morphology, degree of polymerization (DP) and partially digested states.

Finally, our model only simulates the degradation of a single cellulose crystal layer, a feature that should be extended to capture the degradation of a whole cellulose crystal. The major effect from a three-dimensional model is expected to be that the substrate would not be completely accessible at the same time for the cellulases to digest. Also, such a three-dimensional model can capture the possibility that floating sheets of detached substrate may slow cellulases from reaching a larger surface where more efficient digestion is possible. The current model does not account for surface diffusion, which is likely to be important based on results reported by Jervis and colleagues [53], who showed that diffusion does not limit enzyme activity. Fortunately, these deficiencies are not of a fundamental nature because our model is easily extendable and can incorporate them as well as additional properties of various cellulase systems on different types of cellulose surfaces. Importantly, this approach could be broadened to other classes of cellulases and even to cellulosomes as additional experimental data becomes available. For this reason we believe that this model constitutes a significant contribution to the ability to simulate the complicated reactions involved in cellulose degradation.

## Endnotes

^a^Each row corresponds to one of the seven parameters characterizing one monomer while the columns represent numerical values the parameters can take. Each entry of the table denotes a distinct condition of a glucose unit. For a detailed explanation, please see text. ^b^See reference [50]. ^c^See reference [22]. ^d^See reference [49].

## Abbreviations

MD: Molecular dynamics; EG: Endoglucanase; CBH: Cellobiohydrolase; Exo-N: Exocellulase that processes from nonreducing end; Exo-R: Exocellulase that processes from reducing end; Endo: Endoglucanase; NE: Nonreducing end; RE: Reducing end.

## Competing interests

The authors declare that they have no competing interests.

## Authors’ contributions

AA carried out the design, coding and analysis of the approach, and drafted the manuscript. MD aided in efficient implementation of code in different computer platforms. AS aided in verification of the results and literature. TS contributed to design and visual representation of spatial units. PL, AR and SG conceived of the study, participated in its design, verified the results and helped to draft the manuscript. All authors read and approved the final manuscript.
